# Association of Metal Cations with the Anti-PF4/Heparin Antibody Response in Heparin-Induced Thrombocytopenia

**DOI:** 10.21203/rs.3.rs-4385055/v1

**Published:** 2024-05-16

**Authors:** Jason B Giles, Kiana L Martinez, Heidi E Steiner, Andrew Klein, Aikseng Ooi, Julie Pryor, Nancy Sweitzer, Deborah Fuchs, Jason H Karnes

**Affiliations:** University of Colorado Anschutz Medical Campus; University of Arizona College of Pharmacy; University of Arizona College of Pharmacy; University of Arizona College of Pharmacy; University of Arizona College of Pharmacy; Banner University Medical Center-Tucson; Washington University in St. Louis; Banner University Medical Center-Tucson; University of Arizona College of Pharmacy

**Keywords:** anti-PF4/heparin antibodies, heparin-induced thrombocytopenia, ICP-MS, cations, risk factors

## Abstract

Heparin-induced thrombocytopenia (HIT) is an antibody-mediated immune response against complexes of heparin and platelet factor 4 (PF4). The electrostatic interaction between heparin and PF4 is critical for the anti-PF4/heparin antibody response seen in HIT. The binding of metal cations to heparin induces conformational changes and charge neutralization of the heparin molecule, and cation-heparin binding can modulate the specificity and affinity for heparin-binding partners. However, the effects of metal cation binding to heparin in the context of anti-PF4/heparin antibody response have not been determined. Here, we utilized inductively coupled plasma mass spectrometry (ICP-MS) to quantify 16 metal cations in patient plasma and tested for correlation with anti-PF4/heparin IgG levels and platelet count after clinical suspicion of HIT in a cohort of heparin-treated patients. The average age of the cohort (n = 32) was 60.53 (SD = 14.31) years old, had a mean anti-PF4/heparin antibody optical density [OD_405_] of 0.93 (SD = 1.21) units and was primarily female (n = 23). Patients with positive anti-PF4/heparin antibody test results (OD_405_ ≥ 0.5 units) were younger, had increased weight and BMI, and were more likely to have a positive serotonin release assay (SRA) result compared to antibody negative patients. We observed statistical differences between antibody positive and negative groups for sodium and aluminum and significant correlations of anti-PF4/heparin antibody levels with sodium and silver. While differences in sodium concentrations were associated with antibody positive status and correlated with antibody levels, no replication was performed. Additional studies are warranted to confirm our observed association, including *in vitro* binding studies and larger observational cohorts.

## INTRODUCTION

Heparin-induced thrombocytopenia (HIT) is a potentially catastrophic adverse drug reaction (ADR) to the heparin anticoagulants. Heparin is widely prescribed with approximately 1/3 of hospitalized patients receiving the drug and up to 3% of heparin-treated patients developing HIT[1]. HIT is an immune mediated disorder that occurs when immunoglobulin G (IgG) antibodies recognize a neoepitope generated by complexes of heparin bound to endogenous platelet factor 4 (PF4)[2–4]. Complexes of IgG-PF4/heparin bind to receptors present on platelets (as well as neutrophils and monocytes), leading to platelet activation, aggregation and potentially thrombocytopenia and/or thromboembolic complications[5–7].

Factors influencing the risk of HIT have been of great interesting in the field, as treatment for HIT is only actionable after the manifestation of symptoms[8]. Studies have shown genetic polymorphisms alter HIT risk[9–13] with the most confident association seen in HIT associated thrombosis[14]. Other factors such as clinical setting[15], heparin formulation[16], gender[17, 18], and anti-PF4/heparin antibody titers[18] have shown associations with HIT, but many risk factors show only modest increases in chances for progression to full-blown HIT. A key precursor for HIT is the formation of PF4/heparin immune complexes that are ultimately recognized by so called “HIT” antibodies, leading to persistent low platelet count (thrombocytopenia) and/or thromboembolic complications (thrombosis). Research into the early stages leading up to these physical manifestations of HIT may provide additional insights and potentially new therapies that can block these precursor events necessary for HIT to occur.

The binding of PF4 and heparin is a critical step for the antibody response responsible for the HIT phenotype. Without complex formation, no neoepitope is formed for anti-PF4/heparin antibody recognition and subsequent immune response seen in HIT[19, 20]. Complex formation is contingent on numerous factors including proper stoichiometric ratios of PF4 and heparin and the length of the heparin molecule[19]. The anionic charge density of the heparin molecule, necessary for the electrostatic interaction with PF4, can be altered by the binding of metal cations to heparin[21–23]. Additionally, cations including zinc (Zn^2+^) and calcium (Ca^2+^), have been shown to play critical roles in platelet activation, aggregation and ultimately thrombus formation[24–28]. For example, Zn^2+^, in a dose-dependent manner, promotes the binding of heparin to fibrinogen, reducing heparin’s anticoagulant activity[29]. Furthermore, murine knockout models of zinc transporters exhibit hyperreactivity of platelets and enhanced platelet aggregation upon stimuli[30], however, the influence of cations in the context of HIT, specifically the influence of cation plasma concentrations in the anti-PF4/heparin antibody response, is unknown.

We hypothesize dysregulation in circulating cation concentrations could modify the propensity of PF4/heparin complex formation and subsequent antibody response seen in heparin-treated patients. Specifically, a reduction in plasma zinc levels would lower the amount of labile zinc able to neutralize heparin, allowing for charged heparin to bind to PF4 more readily via electrostatic forces. To investigate this potential influence of metal cations in heparin-treated patients, we tested for association and correlation of plasma cation concentrations with markers of anti-PF4/heparin antibody production, including antibody titers and extent of thrombocytopenia. We focus particularly on association and correlation of zinc levels based on the above literature.

## METHODS AND MATERIALS

### Study Population

Suspected HIT patients were recruited from Banner University Medical Center-Tucson (BUMC-T). Recruitment was facilitated through a collaboration with the Sarver Heart Center (Tucson, AZ) and BUMC-T Coagulation Laboratory, which tests for anti-PF4/heparin antibodies in house via enzyme linked immunosorbent assay (ELISA). This study recruited patients clinically suspected to have HIT that received unfractionated heparin or low molecular weight heparin, underwent anti-PF4/heparin antibody testing and were 18 years of age or older at time of enrollment. Patients that tested positive for anti-PF4/heparin antibodies (ELISA OD_405_ ≥ 0.5) were tested for HIT via functional assay (serotonin release assay [SRA]) only if ordered by a provider. Exclusion criteria included pregnancy, patients with hemoglobin less than 9.0 mg/dL, and human immunodeficiency virus (HIV) diagnosis. Patients were recruited from May 2016 to February 2020 and enrollment consisted of a single visit in which a blood draw was performed. This study was approved by the internal review board (IRB) at the University of Arizona. All participants gave informed consent prior to study procedures and sample collection. Subjects consent was obtained in accordance with the Declaration of Helsinki.

Clinical and demographic data was extracted from electronic medical records (EMR) for individuals enrolled in the study. Demographic data, including sex and age, and anthropomorphic measurements such as height, weight and body mass index were obtained from the EMR and stored in REDCap (Research Electronic Data Capture). Self-reported race and ethnicity were obtained from forms provided to the participants specifically for this study. Clinical data including ELISA and SRA results (if available), platelet count at heparin initiation, platelet nadir, heparin anticoagulant formulation, thrombotic events, clinical setting, previous heparin exposure, and ABO blood type were collected. ABO blood type was collected for adjustment as a potential confounder based on a recent GWAS study that identified an association between the O blood group and HIT[9]. Additionally, variables comprising the 4T’s scoring system were collected and independently reanalyzed for this study[31, 32]. The four factors comprising the 4T score - platelet fall count, timing of platelet fall, presence of thrombosis and potential other causes for thrombocytopenia – were abstracted from electronic health records by at least two study investigators independently and any disagreement in 4Ts score was reconciled by the Principal Investigator (JHK).

The majority of analyses conducted in this study interrogated association between cations and anti-PF4/heparin antibody response. As such the cohort was divided based on ELISA results into two groups: 1) antibody-positive [OD_405_ ≥ 0.5] patients; and 2) anti-PF4/heparin antibody-negative [OD_405_ < 0.5]. While the focus of the manuscript was the influence of cations on antibody response, a subset of analyses interrogated associations on HIT itself. For these analyses the cohort was divided into 3 groups: 1) anti-PF4/heparin antibody negative patients [OD_405_ < 0.5], 2) antibody positive patients with an optical density between 2.0 and 0.5 units [2.0 > OD_405_ ≥ 0.5] accompanied by a negative SRA (or missing SRA test), and 3) HIT positive individuals who were deemed to have HIT via a positive SRA result and a 4T score of 4 or greater, or a OD_405_ ≥ 2.0 and 4T ≥ 4. This classification is in line with clinical guidelines such as those set forth by the American Society of Hematology[32].

### Laboratory Testing Procedures

Blood samples (n = 33) were collected in 9-mL EDTA blood collection tubes (BD Vacutainer, Becton Dickinson and Company, New Jersey, USA) and were transported on ice for processing. All samples were processed within two hours of blood draw. Samples were centrifuged at 1200g for 10 minutes at room temperature using SepMate PBMC Isolation tubes (StemCell Technologies, Vancouver, Canada) using the manufacturers protocol. Blood plasma was extracted and stored at −80C until bulk processing for inductively coupled plasma mass spectrometry (ICP-MS).

ELISAs for anti-PF4/heparin antibodies were performed by the Coagulation Laboratory at BUMC-T. Patient plasma was incubated in microwells coated with platelet factor 4 (PF4) complexed to polyvinyl sulfonate (PVS) substrate (Immucor, Waukesha, WI, USA) for detection of anti-PF4/heparin antibodies. An alkaline phosphatase labeled anti-human globulin reagent (anti-IgG) is added to the wells and incubated. Unbound anti-IgG is washed away. A substrate for the alkaline phosphatase (p-nitrophenyl phosphate) is then added and after a 30-minute incubation, the reaction is stopped. Colorimetric change was measured using a spectrophotometer and a negative optical density (OD) result was set at OD_405_ < 0.5. A heparin confirmatory step was used to confirm if a positive screening result was caused by heparin-dependent antibodies. In this step, excess heparin was added to patient plasma prior to test inhibition of the reaction between heparin-dependent antibodies and the PF4/PVS complex.

SRAs were performed by Quest Diagnostics Nichols Institute (San Juan Capistrano, CA). The SRA test is a functional assay that measures heparin-dependent platelet activation and was only performed if ordered by a provider to confirm HIT after a positive ELISA. Patient serum was incubated with donor platelets containing radioactive serotonin (C^14^) and low-dose and high-dose concentrations of heparin. Antibodies present in the patient serum bind and activate donor platelets, releasing radiolabeled serotonin from the platelet granules. A positive SRA was defined as > 20% release of the C^14^ serotonin when mixed with patient serum and low-dose heparin. In addition, a high-dose heparin step was performed to test the platelet activation is heparin dependent. Afifty percent (50%) reduction in activation relative to the low-dose step was used as the cutoff for confirmation.

### Inductively coupled plasma mass spectrometry (ICP-MS)

An Elan DRC-II inductively coupled plasma mass spectrometer was used for the determination of elements in plasma samples. Samples were analyzed at the Arizona Laboratory for Emerging Contaminants center (ALEC, Tucson, AZ). Samples were prepared in accordance with a previously described protocol[33]. Briefly, all samples were homogenized on a Ratek roller mixer. An aliquot of 0.25 mL of each sample was added to a 10 mL acid washed tube with 0.5 mL alkaline solution. Frozen certified reference material were used as quality controls in the ICP-MS analysis of plasma samples as described[33]. In lieu of additional whole blood for use as the standard as described, a synthetic matrix was used for all work at the ALEC laboratory. ICP-MS measured all cations as micrograms per gram of plasma (μg/g) and included the following metal cations; K, Ca, Na, Mg, Be, Al, Mn, Ni, Cu, Zn, Mo, Ag, Cd, Sb, Ba, and Pb.

### Statistical analyses

Data were analyzed using the R programming language (4.2.1). ICP-MS data is presented as micrograms per gram of plasma and mean and standard deviation (SD) were determined for each cation. Data distribution was evaluated using the Shapiro–Wilk test for normality. Basic demographics data is presented as mean and standard deviation (SD), and differences among groups were analyzed using chi-squared tests for categorical variables and one-way ANOVA for continuous variables if data was normally distributed. Otherwise, the non-parametric equivalent Kruskal-Wallis was implemented. The Kruskal-Wallis test was used for determining potential differences in metal cation concentrations between antibody negative and antibody positive groups. Additionally, Kruskal-Wallis was performed in a 3-group comparison as described in the study population section above: 1) antibody negative, 2) antibody positive and 3) HIT positive groups. Correlation between metal cations and anti-PF4/heparin antibodies were tested using Spearman rank correlation.

As is standard in ICP-MS analysis, certain trace elements were ‘not detected’ in a fraction of the samples due to concentrations being lower than the lower limit of quantification (LOQ) for the instrument. These undetected elements were coded as 0 (zero) in all analyses. Correlation between clinical features of thrombocytopenia and cations were interrogated via two variables, proportional platelet drop and absolute platelet nadir values. The variable “proportional platelet drop” was produced by calculating the proportional difference between platelet count at heparin initiation and platelet nadir (platelet high – nadir) / platelet high. Statistically significant associations and correlations were determined by alpha (α) < 0.05.

Other studies have shown, *in vivo*, physiologically relevant cations may act in concert with each other in a synergistic manner to affect the binding of heparin to heparin-binding partners[23, 34]. To interrogate the effects of varying mixed cation concentrations on HIT progression we performed a Principal Components Analysis (PCA)[35–37]. In short, PCA is a multivariate technique that extracts the critical information from a set of variables, reduces the dimensionality of the data, and presents a new set of variables, called principal components (PCs), which represent as much of the original information as possible in as few variables as possible. PCA was performed using the *prcomp* package in R, where the package centered and scaled the data in a pre-processing step, as is standard practice in PCA[38, 39]. Contributions of the response variables (individual cations) to principal components (PCs) were calculated by the squared cosines of the variables and visualized via a loading plot of vectors for each variable. Testing of statistical differences between groups using PC was performed via a Permutational Multivariate Analysis of Variance (PerMANOVA) test.

## RESULTS

A total of 33 patients were recruited, but one individual was removed entirely from analyses. This individual was considered “HIT positive” based on ELISA results (OD = 2.89), but had a 4T score of 1 and no subsequent SRA test, resulting in a clinical picture incompatible with HIT. After removal, a total of 32 suspected HIT patients were included in our study. The average [SD] age was 60.53 [14.31] years with 23 female patients (71.9%). Mean [SD] anti-PF4/heparin antibody optical density (OD_405_) was 0.93 [1.21] units (Table 1). Mean platelet high count was 218.12×10^3^ (130.25) platelets/μL with an average platelet nadir of 62.47×10^3^ (37.56) platelets/μL. On average, platelet nadir was observed 9.58 [9.2] days after the initiation of heparin therapy. All patients received unfractionated heparin (UFH) and 9 patients also received enoxaparin in addition to UFH. The race/ethnicity of the cohort consisted of 29 white individuals, 1 African American and 2 non-responders with 9 patients (28.1%) being of Hispanic or Latino ethnicity. Patients were largely admitted into either cardiovascular (n = 13) or Intensive Care Unit (n = 6) settings. The ABO O-blood group represented the highest frequency of participants with 14 total, followed by A (n = 10), B (n = 1) and AB (n = 1) groups. Summary statistics for clinical progression and severity (functional assay [SRA], and thrombotic events) as well as anthropometric traits such as height, weight, and body mass index (BMI) are listed in Table 1.

Stratification of patients classified as either anti-PF4/heparin antibody positive [OD_405_ ≥ 0.5]), or negative [OD_405_ < 0.5], resulted in statistically significant differences in age, weight, BMI, and SRA status (Table 1). Patients with positive anti-PF4/heparin antibodies were significantly younger (51.16 [SD = 14.48] vs. 65.44 [11.79] years of age, p = 0.005), had increased weight (110.95 [30.52] kg vs. 77.99 [16.44] kg, p < 0.001) and subsequently higher BMI (36.75 [8.93] kg/ m^2^ vs. 27.52 [5.86] kg/m^2^, p = 0.001), and were more likely to be diagnosed with HIT (6 [54.5%] vs. 0, p < 0.001). Those individuals that tested positive for anti-PF4/heparin antibodies received ELISA results 10.4 [7.26] days after heparin administration, compared to 8.8 [10.87] days for anti-PF4/heparin antibody negative patients (p = 0.68). Among the three groups, there was a statistically significant difference in mean age, antibody OD units, weight, BMI, and proportion of patients with thrombosis (Table 2).

**Figure 1:**
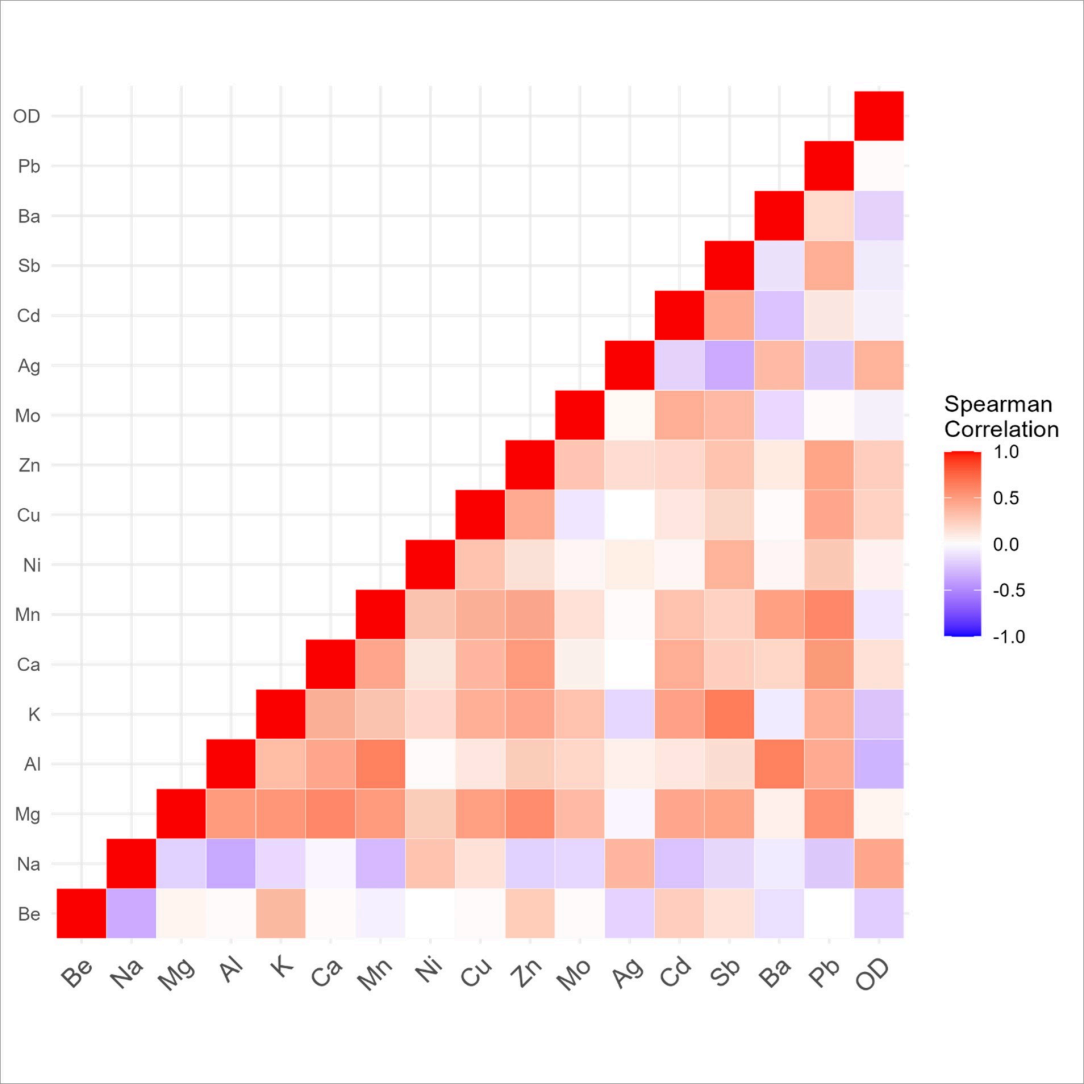
Correlation plot of anti-PF4/heparin antibodies and metal cations. The ‘OD’ variable denotes anti-PF4/heparin optical density units readout from ELISAs. Figure legend (right) indicates increasing opacity corresponds to Spearman correlation coefficients (Rho [ρ]) further from zero. Red squares show direct correlation and blue squares indicate inverse correlation between the two variables. Be: Beryllium, Na: Sodium, Mg: magnesium, Al: Aluminum, Ca: Calcium, Mn: Manganese, Ni: Nickel, Cu: Copper, Zn: Zinc, Mo: molybdenum, Ag: Silver, Cd: Cadmium, Sb: Antimony, Ba: Barium, Pb: Lead

Shapiro Wilks testing indicated that OD values and all cations were non-normal in their distribution. The top five most abundant cations in plasma included sodium (Na), potassium (K), calcium (Ca), magnesium (Mg), and zinc (Zn). Table 3 shows the mean [SD] cation concentrations for the cohort. Sodium, the most abundant cation in the cohort, showed a statistically significant difference (Table 3) between antibody positive and negative groups (p = 0.037, negative = 2.754×10^3^ (7.449×10^2^), positive = 3.746×10^3^ (1.794×10^3^)). Aluminum also showed statistical differences between the two groups (p = 0.049, negative = 1.941×10^− 1^ (1.816×10^− 1^), positive = 1.271×10^− 1^ (1.41×10^− 2^). No cations showed statistically significant differences between the three groups. Correlation across all cations and antibody OD units are presented in a heat map (Fig. 1). The spearman test showed two metal cations were statistically correlated with anti-PF4/heparin antibody levels (Table 4). Sodium, the most abundant cation in plasma/serum for the cohort, was significantly correlated with increasing anti-PF4/heparin antibody OD units (rho [ρ] = 0.44, p = 0.011). Silver (Ag) was also significantly correlated with anti-PF4/heparin antibodies (ρ = 0.38, p = 0.03).

**Figure 2:**
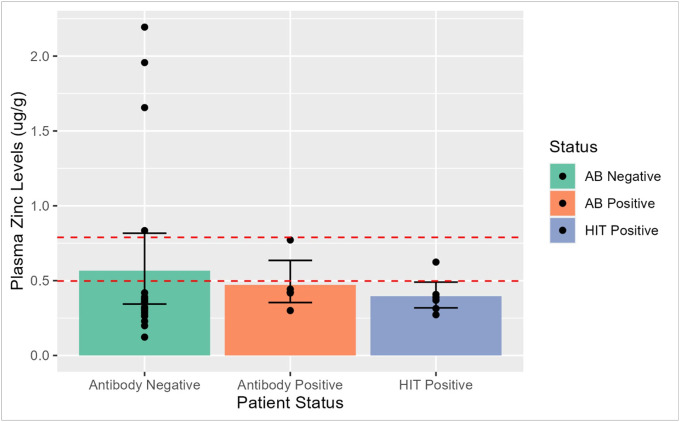
Box & Whisker plot with accompanying scatterplot showing distribution of zinc concentrations. Plot shows the cation concentrations (μg/g) for zinc (Y-axis) across the three cohorts: 1) antibody negative (green), 2) antibody positive/HIT negative (orange) and 3) HIT positive groups (blue). HIT positivity was determined via serotonin release assay [SRA] and a 4T score of 4+. Red dotted lines indicate physiological range (5%−95% CI) for plasma zinc concentration Figure legend (right) indicates group status. AB: anti-PF4/heparin antibody; HIT: heparin-induced thrombocytopenia.

In order to further investigate the influence of zinc in HIT pathogenesis, we performed a Fisher Exact test between antibody negative and antibody positive individuals to determine association with physiological normal range for zinc[40]. No significant differences in counts between the two groups was observed (p = 0.27) (Fig. 2).

**Figure 3:**
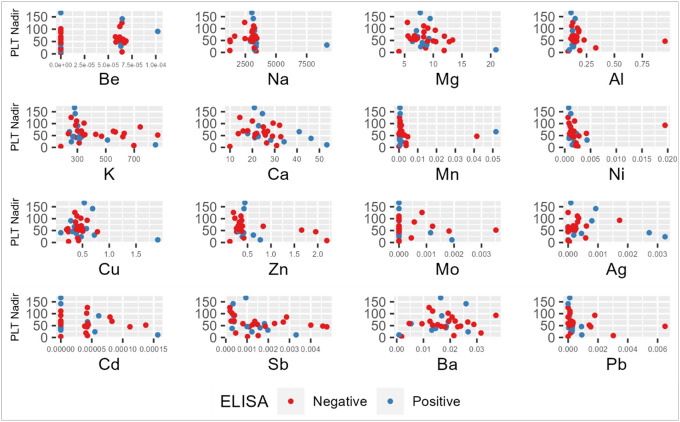
Scatterplot of platelet nadir (1×10^3^ platelets/microliter) and metal cations. Scatterplot shows the distribution of cation concentrations (μg/g) against platelet nadir for each individual in the cohort. Additionally, plot depicts individuals based on ELISA results, colored in blue for antibody ‘positive’ (OD_405_ ≥ 0.5 units) patients or in red for antibody ‘negative’ individuals (OD_405_< 0.5 units). PLT: platelet, Be: Beryllium, Na: Sodium, Mg: magnesium, Al: Aluminum, Ca: Calcium, Mn: Manganese, Ni: Nickel, Cu: Copper, Zn: Zinc, Mo: molybdenum, Ag: Silver, Cd: Cadmium, Sb: Antimony, Ba: Barium, Pb: Lead.

**Figure 4:**
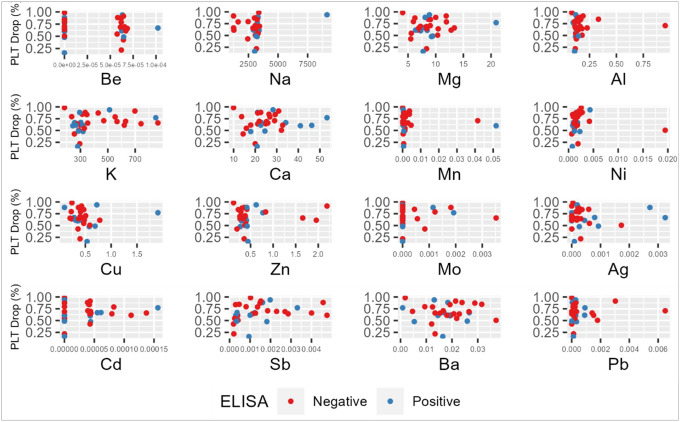
Scatterplot of proportional platelet drop and metal cation concentrations. Scatterplot shows the distribution of cation concentrations (μg/g) against platelet drop, from heparin initiation to platelet nadir, for each individual in the cohort. Additionally, plot depicts individuals based on ELISA results, colored in blue for antibody ‘positive’ (OD_405_ ≥ 0.5 units) patients or red for antibody ‘negative’ individuals (OD_405_< 0.5 units). PLT: Platelet, Be: Beryllium, Na: Sodium, Mg: magnesium, Al: Aluminum, Ca: Calcium, Mn: Manganese, Ni: Nickel, Cu: Copper, Zn: Zinc, Mo: molybdenum, Ag: Silver, Cd: Cadmium, Sb: Antimony, Ba: Barium, Pb: Lead.

Correlation between platelet activation and cations were interrogated via two additional variables, proportional platelet drop and absolute platelet nadir values (Table 4). Three cations – antimony (Sb), nickel (Ni) and lead (Pb) – were statistically correlated with platelet nadir within our patient cohort. Figure 3 shows platelet nadir distributions for each cation among anti-PF4/heparin antibody positive and antibody negative individuals. The proportional platelet drop, or percent change, from platelet count high to nadir showed statistical correlation with antimony and nickel as was observed with spearman correlation of absolute platelet counts (Fig. 4 & Table 4).

To interrogate the potential effects of mixed cation concentrations on antibody response, we performed PCA. The largest contributing cations - Magnesium (Mg), potassium (K), and cadmium (Cd) – showed near equal percent contribution (~ 15% each), followed by antimony (Sb) (11%) and zinc (10%) in the PCA. These top contributors to the PCs also showed high correlation with each other based on the directionality of their vectors.

**Figure 5:**
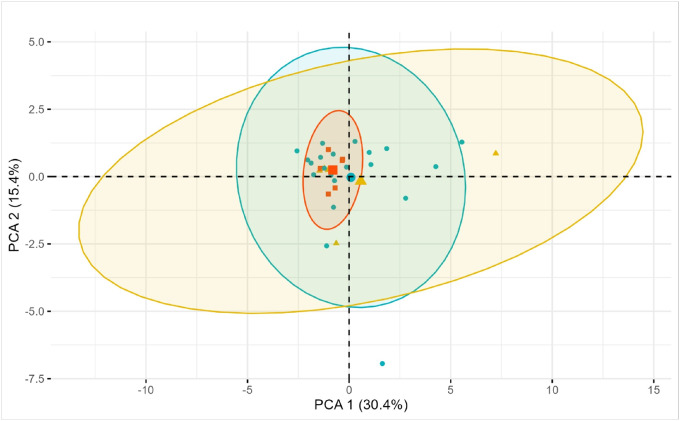
Principal Component Analysis (PCA) plot by group. Individuals in the cohort are shown plotted by their values for PC1 (X-axis) and PC2 (Y-axis). Colors and shapes represent the three groups each individual is assigned to: Antibody negative (green circle), Antibody positive (yellow triangle) or HIT positive (red square). Corresponding colored ellipses represent T-distribution where 95% of the data points are expected to be included based on the current sample. The center of each group ellipse is denoted by a single larger point corresponding to each colored ellipse (Antibody negative - green circle, Antibody positive - yellow triangle and HIT positive - red square).

Individuals were first grouped by status (antibody negative, antibody positive or HIT positive) and visualized via PC1 and PC2, which explained 30.4% and 15.4% of the variability in the data, respectively (Fig. 5). While groups overlapped in their inclusion bounds (ellipses of data points), the HIT positive individuals’ ellipse was much tighter (red ellipse – Fig. 5) than the antibody positive or antibody negative groups. To analyze if these groups were different, we performed a PerMANOVA test on the clusters. Despite the visual differences among the groups, the analysis indicated the group clusters were not statistically different (p = 0.213). This indicates a collection of cations, as represented by PCs, were not informative enough to accurately classify our patients into their respective groups (antibody negative, antibody positive or HIT positive).

## DISCUSSION

In this study we investigated the association between plasma cation concentrations and the antibody response of HIT. Given that PF4/heparin complexes are necessary for neoepitope formation and subsequent recognition by anti-PF4/heparin IgG antibodies for HIT to occur, perturbations in complex formation may have effect on the number of neoepitope sites present for antibody binding, potentially reducing the severity of the antibody response seen in HIT. To test the potential influence of cations on complex formation, we interrogate the association between cations and extent of platelet activation. Differences in mean ranks via Kruskal-Wallis were observed between antibody positive and antibody negative patients for sodium and aluminum. Sodium, the most abundant cation in plasma for the cohort, was also significantly correlated with increasing anti-PF4/heparin antibody OD units.

Given prior evidence, we hypothesized zinc may influence the binding of heparin to PF4, as has been observed with heparin and other heparin binding partners[28]. Zinc increases the affinity of brinogen for heparin, sequestering heparin and reducing its anticoagulant capability[29]. Zinc, in a dose-dependent manner, also augments the binding of heparin to histidine-rich glycoprotein, restricting the ability of heparin to bind to antithrombin to exert its anticoagulant properties[41]. In this study, we compared endogenous cation concentrations across the three groups and did not observe differences in mean cation concentrations among the three groups in our cohort.

Under physiological conditions, combination of cations act in concert to impact the interaction of heparin with binding partners[23]. To investigate this synergistic effect in the context of HIT, we performed PCA analysis. PerMANOVA testing showed that the first 2 principal components are not informative enough to differentiate the groups in our population. This indicates that a collection of cations does not affect the antibody response in a statistically observably manner.

Beyond the influence of cations on HIT pathogenesis, previous work has identified several clinical/demographic variables as risk factors for increasing anti-PF4/heparin antibodies including BMI, diabetes, and surgery circumstances[42–44]. In this study, a number of demographic variables were associated with positive anti-PF4/heparin antibody ELISA results. Patients that possessed anti-PF4/heparin antibodies were younger, had increased weight, and higher BMI. These results, particularly higher BMI, are in line with Warkentin *et al*[42], who found greater risk of anti-PF4/heparin antibody immune response with higher BMI quantiles in enoxaparin-treated patients. These associations were identified in a pool of patients all receiving unfractionated heparin and despite the increase of detectable levels of anti-PF4/heparin IgG antibodies in UFH patients[44], the clinical/demographic variables were still significant in our cohort. The replicated associations of previously identified demographic variables (age, BMI/weight) indicate additional factors, beyond heparin type, increase the risk of anti-PF4/heparin antibody response and subsequently HIT risk in heparin-treated patients.

While a number of statistically significant observations were observed in our cohort, including replication of previously observed associations of clinical features, there are several limitations worthy of mention in our study. The small sample size likely resulted in a reduced power to observe even modest effect sizes within our cohort. We also did not implement a multiple comparisons adjustment. Timing of HIT-related tests compared to blood sampling may have confounded our results. Recruitment of patients followed ELISA results and changes in plasma cation concentrations may be altered leading up to ELISA or platelet nadir but returned to homeostasis before biospecimens were able to be collected. ELISA results were presented in terms of optical density units instead of using a standard curve to convert the OD values into more standardized, meaningful units. Within the clinical, OD units are almost universally used to aid in HIT diagnosis with no conversion via a standard curve[18], but using these standardized units would enhance comparability across assays. Finally, the generalizability of our results may be limited since recruitment took place at a single hospital and our population was largely confined to non-Hispanic white females.

## CONCLUSION

We identified associations with demographic variables (age, weight, and BMI) and anti-PF4/heparin antibody response in a cohort of heparin-treated patients suspected of HIT. We replicated previous signals including the association between increasing BMI and antibody OD units. We observed statistical differences between antibody positive and negative groups for sodium and aluminum and significant correlations with PF4/heparin antibody levels with sodium and silver. No other cations, including zinc, were significantly associated nor correlated with PF4/heparin antibody levels. However, given the lack of replication and small sample size, additional studies are warranted to identify association between anti-PF4/heparin antibody response and metal cation concentrations.

## Figures and Tables

**Table 1:** Demographic and Laboratory Characteristics of the Cohort

Trait	All Patients	Anti-PF4/heparin antibody negative	Anti-PF4/heparin antibody positive^1^	P-value^2^
Total, n (%)	32	21	11	-
Age (years)	60.53 (14.31)	65.44 (11.79)	51.16 (14.48)	0.005
Sex, female n (%)	23 (71.9)	13 (61.9)	10 (90.9)	0.187
Anti-PF4/heparin IgG OD^3^	0.93 (1.21)	0.11 (0.10)	2.33 (0.87)	< 0.001
Time to ELISA (from heparin initiation)	7.0 (6.09) days	8.81 (10.87)	10.40 (7.26)	0.679
Time to platelet nadir (from heparin initiation)	7.2 (5.70) days	9.67 (10.39)	9.40 (6.48)	0.941
Height (cm)	170.21 (8.99)	168.41 (7.59)	173.66 (10.76)	0.118
Weight (Kg)	89.32 (26.98)	77.99 (16.44)	110.95 (30.52)	< 0.001
Body mass index (kg/m^2^)	30.69 (8.23)	27.52 (5.86)	36.75 (8.93)	0.001
Highest platelet count (1×10^3^ platelets/microliter)	218.12 (130.25)	226.86 (128.47)	201.45 (138.27)	0.608
Platelet nadir (1×10^3^ platelets/microliter)	62.47 (37.56)	61.14 (30.94)	65.00 (49.48)	0.788
Thrombosis (venous or arterial), n (%)	10 (30.3)	4 (19.0)	6 (54.6)	0.081
**Functional Assay (SRA) [physician ordered]**	14 (42.42%)	6 (28.57%)	8 (72.7%)	0.077
*SRA positive*, n (%)	3	0 (0.0)	3 (27.3%)	0.023
**4T Score**				0.193
*Low Risk (0–3)*	14 (49.9)	11 (64.7)	3 (27.3)	
*Intermediate Risk (4–5)*	13 (44.8)	6 (35.2)	7 (63.6)	
*High Risk (6–8)*	1 (3.4)	0 (0.0)	1 (9.1)	
**Clinical setting**, n (%)		-		0.977
*Cardiovascular*	13 (43.3)	8 (42.1)	5 (45.5)	-
*Intensive care unit*	6 (19.4)	4 (21.1)	2 (18.2)	-
*Unknown*	11 (35.5)	7 (36.8)	4 (36.4)	-
**ABO**, n (%)		-		0.459
*Type A*	10 (38.5)	5 (33.3)	5 (45.5)	-
*Type AB*	1 (3.8)	0 (0.0)	1 (9.1)	-
*Type B*	1 (3.8)	1 (6.7)	0 (0.0)	-
*Type O*	14 (53.8)	9 (60.0)	5 (45.5)	-

Values represent mean [standard deviation] unless otherwise specified; n [%]: Total count in each category and the percentage of the overall sample size. PF4/heparin antibody levels were determined using enzyme-linked immunosorbent assay (ELISA). OD indicates optical density; SRA: serotonin release assay.

1 Anti-PF4/heparin antibody positive patients are all individuals who have an ELISA OD_405_ ≥ 0.5 regardless of HIT determination. OD_405_ < 0.5 units are classified as controls.

2 P-values listed are from comparison of the trait between antibody positive and negative patients. Differences between groups were analyzed using chi-squared tests for categorical variables and one-way ANOVA for continuous variables or non-parametric equivalents (Kruskal-Wallis) if data was not normally distributed.

3 Anti-PF4/heparin antibody levels were determined using enzyme-linked immunosorbent assay (ELISA)

**Table 2:** Cohort Demographic and Laboratory Characteristics by Study Group^1^

Trait	Antibody Negative	Antibody Positive (HIT Negative)	HIT Positive	P-value^2^
Total, n (%)	21	5	6	
Age (years)	65.44 (11.79)	50.20 (13.62)	51.96 (16.42)	0.021
Sex, female n (%)	13 (61.9)	4 (80.0)	6 (100.0)	0.17
Anti-PF4/heparin IgG OD^3^	0.11 (0.10)	1.77 (0.82)	2.79 (0.62)	< 0.001
Time to ELISA (from heparin initiation)	8.81 (10.87)	15.80 (4.87)	5.00 (4.69)	0.201
Time to platelet nadir (from heparin initiation)	9.67 (10.39)	13.00 (6.78)	5.80 (4.02)	0.479
Height (cm)	168.41 (7.59)	172.88 (14.75)	174.31 (7.50)	0.291
Weight (Kg)	77.99 (16.44)	123.94 (38.19)	100.12 (19.67)	< 0.001
Body mass index (kg/m^2^)	27.52 (5.86)	41.04 (9.11)	33.18 (7.71)	0.001
Highest platelet count (1×10^3^ platelets/microliter)	226.86 (128.47)	207.80 (114.52)	196.17 (166.35)	0.87
Platelet nadir (1×10^3^ platelets/microliter)	61.14 (30.94)	85.40 (66.61)	48.00 (24.16)	0.256
Thrombosis (venous or arterial), n (%)	4 (19.0)	2 (40.0)	4 (66.7)	0.028
**4T Score** ^4^				0.213
*Low Risk (0–3)*	11 (64.7)	3 (60.0)	0 (0.0)	
*Intermediate Risk (4–5)*	6 (28.6)	2 (40.0)	6 (83.2)	
*High Risk (6–8)*	0 (0.0)	0 (0.0)	1 (16.7)	

Values represent mean (standard deviation) unless otherwise specified; n (%): Total count in each category and the percentage of the overall sample size. OD indicates optical density; Kg = kilograms; m (m^2^) = meters; cm = centimeters.

1 Anti-PF4/heparin antibody negative [ELISA < 0.5], anti-PF/heparin antibody positive [ELISA > 0.5], heparin-induced thrombocytopenia (HIT) positive.

2 P-values listed are from comparison of the trait between groups. Differences between groups were analyzed using one-way ANOVA for continuous variables or non-parametric equivalents (Kruskal-Wallis) if data was not normally distributed.

3 Anti-PF4/heparin antibody levels were determined using enzyme-linked immunosorbent assay (ELISA)

4 The four factors comprising the 4T score - platelet fall count, timing of platelet fall, presence of thrombosis and potential other causes for thrombocytopenia – were abstracted from electronic health records.

**Table 3:** Inductively couples plasma mass spectrometry (ICP-MS) summary data

Cation	Mean fog/g) [Standard Deviation (SD)]	Controls (anti-PF4/heparin antibody negative)^1^	Anti-PF4/heparin antibody positive^1^	P-value^2^
Ag	4.357×10^−4^ (7.61×10^−4^)	2.505×10^−4^ (3.857×10^−4^)	7.892×10^−4^ (1.136×10^−4^)	0.227
Al	1.711×10^−1^ (1.49×10^−1^)	1.941×10^−1^ (1.816×10^−1^)	1.271×10^−1^ (1.41×10^−2^)	0.049
Ba	1.771×10^−2^ (8.232×10^−3^)	1.861×10^−2^ (8.544×10^−3^)	1.60×10^−2^ (7.690×10^−3^)	0.463
Be	3.29×10^−5^ (3.41×10^−5^)	3.62×10^−5^ (3.22×10^−5^)	2.66×10^−5^ (3.84×10^−5^)	0.497
**Ca**	25.64 (9.048)	23.22 (6.283)	30.246 (11.80)	0.226
Cd	3.59×10^−5^ (4.18×10^−5^)	3.75×10^−5^ (3.92×10^−5^)	3.27×10^−5^ (4.83×10^−5^)	0.820
Cu	4.680×10^−1^ (3.016×10^−1^)	4.120×10^−1^ (1.302×10^−1^)	5.748×10^−1^ (4.785×10^−1^)	0.463
**K**	4.069×10^2^ (1.870×10^2^)	4.328×10^2^ (1.903×10^2^)	3.574×10^2^ (1.785×10^2^)	0.137
**Na**	3.0950×10^3^ (1.275×10^3^)	2.754×10^3^ (7.449×10^2^)	3.746×10^3^ (1.794×10^3^)	0.037
**Mg**	8.815 (3.077)	8.610 (2.563)	9.207 (3.993)	0.984
Mn	3.881×10^−3^ (1.132×10^−3^)	3.178×10^−3^ (8.878×10^−3^)	5.223×10^−3^ (1.540×10^−3^)	0.393
**Mo**	3.586×10^−4^ (7.897×10^−4^)	4.002×10^−4^ (8.679×10^−4^)	2.792×10^−4^ (6.451×10^−4^)	0.602
Ni	2.2018×10^−3^ (3.267×10^−3^)	2.514×10^−3^ (3.967×10^−3^)	1.606×10^−3^ (1.013×10^−3^)	0.488
Pb	6.174×10^−4^ (1.260×10^−3^)	7.831×10^−4^ (1.525×10^−3^)	3.010×10^−4^ (3.178×10^−4^)	0.952
Sb	1.473×10^−3^ (1.280×10^−3^)	1.611×10^−3^ (1.431×10^−3^)	1.210×10^−3^ (9.29×10^−3^)	0.620
Zn	5.193×10^−1^ (4.889×10^−1^)	5.661×10^−1^ (5.942×10^−1^)	4.300×10^−1^ (1.470×10^−1^)	0.211

Values represent mean (standard deviation).

1 PF4/heparin antibody levels were determined using enzyme-linked immunosorbent assay (ELISA). OD_405_ < 0.5 units are classified as controls.

2 P-values listed are from comparison of the cation between cases and controls in the cohort. Differences among groups were analyzed using non-parametric Kruskal-Wallis one-way analysis of variance.

Ag: Silver, Al: Aluminum, Ba: Barium, Be: Beryllium, Ca: Calcium, Cd: Cadmium, Cu: Copper, K: Potassium, Na: Sodium, Ni: Nickel, Mg: Magnesium, Mn: Manganese, Mo: Molybdenum, Pb: Lead, Sb: Antimony, Zn: Zinc

**Table 4:** Spearman Correlation Analysis of Metal Cations with Anti-PF4/heparin OD values, Platelet Nadir, and Proportional Platelet Count Decrease

Outcome	Anti-PF4/heparin Antibody OD_405_		Platelet Nadir		Proportional Platelet Drop^1^	
Cation	Rho [ρ]	P-Value	Rho [ρ]	P-Value	Rho [ρ]	P-Value
Ag	0.3837	0.030	0.211	0.245	−0.055	0.764
Al	−0.316	0.078	−0.007	0.968	0.143	0.433
Ba	−0.185	0.31	0.078	0.672	−0.018	0.923
Be	−0.207	0.256	0.125	0.494	−0.0242	0.895
Ca	0.150	0.414	−0.310	0.085	0.123	0.500
Cd	−0.055	0.765	−0.137	0.455	0.0333	0.856
Cu	0.225	0.216	0.078	0.670	−0.188	0.301
K	−0.248	0.170	−0.159	0.385	0.292	0.104
Na	0.443	0.011	−0.186	0.309	0.155	0.395
Ni	0.059	0.748	−0.378	0.033	0.356	0.046
Mg	0.046	0.801	−0.199	0.274	0.071	0.695
Mn	−0.099	0.589	−0.059	0.749	−0.046	0.803
Mo	−0.053	0.77	−0.119	0.517	0.234	0.197
Pb	0.01	0.926	−0.398	0.024	0.091	0.621
Sb	−0.086	0.641	−0.475	0.006	0.437	0.0130
Zn	0.249	0.170	−0.253	0.162	0.074	0.686

Rho (ρ): Spearman correlation coefficient. OD indicates optical density determined using enzyme-linked immunosorbent assay (ELISA).

1 Proportional Platelet Drop was calculated by taking initial platelet count at time of heparin therapy minus absolute platelet nadir count then divided by initial platelet count.

Ag: Silver, Al: Aluminum, Ba: Barium, Be: Beryllium, Ca: Calcium, Cd: Cadmium, Cu: Copper, K: Potassium, Na: Sodium, Ni: Nickel, Mg: Magnesium, Mn: Manganese, Mo: Molybdenum, Pb: Lead, Sb: Antimony, Zn: Zinc
